# A Practical Route for the Preparation of 1,4,7-Triazacyclononanyl Diacetates with a Hydroxypyridinonate Pendant Arm

**DOI:** 10.3390/molecules201019393

**Published:** 2015-10-23

**Authors:** Yongkang Gai, Zhongping Hu, Zhao Rong, Xiang Ma, Guangya Xiang

**Affiliations:** School of Pharmacy, Tongji Medical College, Huazhong University of Science and Technology, Wuhan 430030, China; E-Mails: gykmail@gmail.com (Y.G.); maxwellsg@163.com (Z.H.); wojiaorz@gmail.com (Z.R.)

**Keywords:** 1,4,7-triazonane, hydroxypyridinonate, metal chelator

## Abstract

The preparation of triazamacrocyclic hydroxypyridinonate (HOPO-TACN) derivatives as potential chelators for metals in biomedical applications was reported. The synthesis is based on a convergent synthetic approach, in which the key intermediate di-*tert*-butyl-2,2′-(1,4,7-triazonane-1,4-diyl) diacetate was coupled with a hydroxypyridinonate pendant arm. The method is suitable for rapid syntheses of metal chelator HOPO-TACNs of biomedical interest.

## 1. Introduction

There has been considerable interest in the synthesis and coordination chemistry of polyazacycles with coordinating pendant arms because of their potential use in applications such as magnetic resonance imaging (MRI) contrast agents [1,2], positron emission tomography (PET) [3,4], luminescence probes [5,6,7,8], catalysis [9], and so on. Among the polyazacycle derivatives, tetraazamacrocycles with acetate side chains have received the most attention owing to their strong chelating properties and higher selectivity toward di- or trivalent metal ions [10,11,12,13]. In contrast to tetraazamacrocycles, less attention has be paid on the triazamacrocycle derivatives [12,14,15,16,17,18,19,20,21]. The interest in the triazamacrocycle derivatives, especially 1,4,7-triazacyclononane (TACN) and TACN-based derivatives, has continued to grow in the past few years in the field of nuclear medicine for diagnosis and therapy [4,22,23,24].

TACN derivatives provide versatile platforms for strongly chelating radiometals such as ^90^Y, ^177^Lu, ^212/213^Bi, ^67/68^Ga, ^64/67^Cu, ^99m^Tc and ^18^F-Al for SPECT, PET imaging and radiotherapy applications [25,26]. The strong coordination capabilities and high selectivity of the TACN derivatives toward di- or trivalent metal ions were determined by the cooperative binding mode from TACN and pendant arms. The pendent arm on TACN platform is expected to accelerate complexation with isotopes while maintaining a high level of stability of the complexes, coupled with the preorganization and macrocyclic effect of the TACN substructure [27]. So far, some researchers have been focused on the TACN derivatives with acetate pendant arms and phosphinate pendant arms. However, far less attention had been paid to other suitable pendent donor groups. In addition, selecting the correct type of chelating unit is one of the most decisive factors in achieving high selectivity toward the specific metal ion.

A class of multidentate hydroxypyridinonate (HOPO) was synthesized and studied for their outstanding abilities to scavenge lanthanide and actinide ions [28]. In particular, the octadentate ligand 3,4,3-LI(1,2-HOPO) has been identified as an effective metal chelator and is currently undergoing development as a prospective therapeutic actinide decorporation agent [29]. The pendent oxygen-containing donor hydroxypyridinonate demonstrated high affinities to hard Lewis acid.

In this context, new prospects for the synthesis of new chelating agents may be delineated while combining geometric and functional features of the TACN platform and the structural potential of common oxygen-containing donor, such as HOPO, and carboxylic acid. A triazamacrocycle with one HOPO and two carboxylic acids is ideally suited to form seven-coordinate complexes with two groups of facial donor, where the macrocyclic nitrogen atoms on the one side and the pendant donors on the other. The lanthanide ions and many other metal ions have coordination numbers larger than six, thus, these ligands have the potential to be excellent chelating agent in the MRI or radioimmunotherapy (RIT) applications.

**Figure 1:**
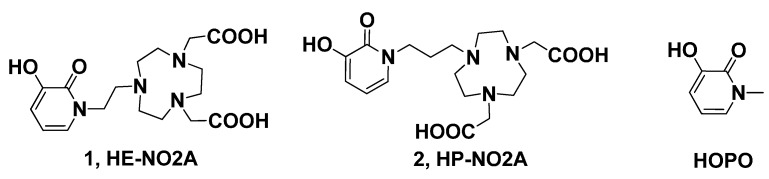
Structures of novel ligands HE-NO2A, HP-NO2A.

With the aim of expanding the diversity of TACN based chelators which may permit orientation for all donors to assume an optimal geometry with coordination sphere, we explored the synthesis of HOPO-TACN derivatives, 2,2ʹ-(7-(2-(3-hydroxy-2-oxopyridin-1(2*H*)-yl)ethyl)-1,4,7-triazonane-1,4-diyl) diacetic acid (**1**, HE-NO2A) and 2,2ʹ-(7-(3-(3-hydroxy-2-oxopyridin-1(2*H*)-yl)propyl)-1,4,7-triazonane-1,4-diyl) diacetic acid (**2**, HP-NO2A) as potential chelators for metal in the biomedical applications (Figure 1).

## 2. Results and Discussion

**Scheme 1:**
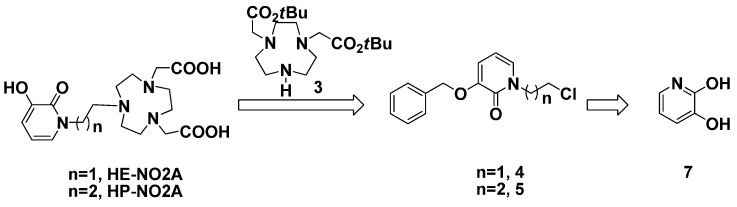
Retrosynthetic route to HE-NO2A and HP-NO2A.

The synthetic strategy for the preparation of the target ligands HE-NO2A and HP-NO2A involves the synthesis and coupling reaction of the fragment **3** with **4** or **5**, which were obtained from readily available starting materials 1,4,7-triazacyclononane (TACN) and 3,2-hydroxypridinone (3,2-HOPO), respectively. The practical, reproducible, and readily scalable synthetic route and efficient purification of all precursor molecules for macrocyclic HOPO-TACN derivatives was developed as shown in Scheme 1.

**Scheme 2:**
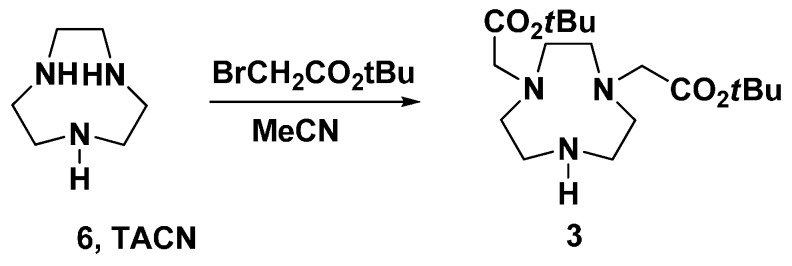
Synthesis of fragment **3**.

Fragment **3** were synthesized from 1,4,7-triazacyclononane (**6**) by reacting with *tert*-butylbromoacetate using the reported methods (Scheme 2) [24,30,31,32]. However, the yield was not good, because the reaction produced a mixture of mono-, di-, and trisubstituted TACN. We then optimized the reaction conditions by choosing a different solvent, optimizing the amount and ratio of reactants, and adding base to scavenge the protons formed during the reaction. In many attempts, we found that adding bases such as potassium carbonate, TEA, or sodium bicarbonate to the reaction negatively affected the yield of disubstituted compounds. The possible reason was that the protons produced *in-situ* would react with the unsubstituted amine to prevent oversubstitution to give trisubstituted compounds. In addition, through optimizing the ratio of reactants, the best yield was obtained when 2.0 eq. of *tert*-butylbromoacetate to TACN was used in the reaction. Finally, using acetonitrile as a solvent to alkylate TACN with 2 eq. *tert*-butyl bromoacetate gave as the major product disubstituted TACN in an acceptable yield as monitored by TLC. While the purification using column chromatography was unsuccessful, a carefully pH-controlled extraction was applied to obtain the fragment **3** in 49% isolated yield.

**Scheme 3:**
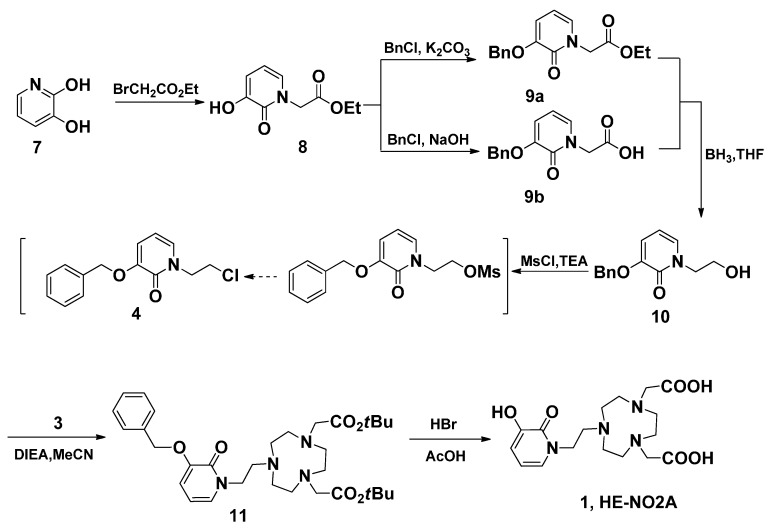
The synthesis of ligand HE-NO2A (**1**).

As shown in Scheme 3, the synthesis of fragment **4** was started from 3,2-hydroxypridinone, which was refluxed with 5 eq. ethyl bromoacetate under the protection of N_2_ to give **8** in 86% yield. In order to get the pure alcohol **10**, the needed **9a** was obtained via the hydroxyl function protection of **8** by *O*-benzylation. However, column chromatography must be applied to purify both **9a** and **10**. In another approach, **9b** was directly obtained by *O*-benzylation and hydrolysis in one batch, and the purification by recrystallization proved very convenient. The following reduction of carboxylic acid **9b** with BH_3_/THF was easily carried out to afforf **10** in higher yield than the approach from **9a**. When we tried to convert alcohol **10** into **10**-OMs by treating it with 1.2 eq. of MsCl in chloroform in the presence of 3 eq. of TEA, an unexpected chloride **4** was obtained. The same product was obtained by treating **10** with TsCl [33]. During the above reaction, two new spots were formed visualized by TLC during the first few hours, which cannot be separated from the reaction mixture. We presumed that the unidentified spot was the intermediate **10**-OMs. This spot was slowly converted into the other, and the whole reaction proceeded efficiently to give the chloride **4** in one batch in 72% yield. With chloride **4** in hand, HE-NO2A (**1**) was prepared readily from disubstituted TACN **3** in two steps (Scheme 3). The alkylation of disubstituted TACN **3** with **4** using DIEA in refluxing MeCN gave the trisubstituted TACN **11** in excellent yield. Further reaction of **11** with the hydrolysis agent HBr/HOAc at room temperature gave the target ligand **1** in 91% yield, where the *O*-benzyl hydroxyl protection functional group was simultaneously removed in the reaction.

**Scheme 4:**
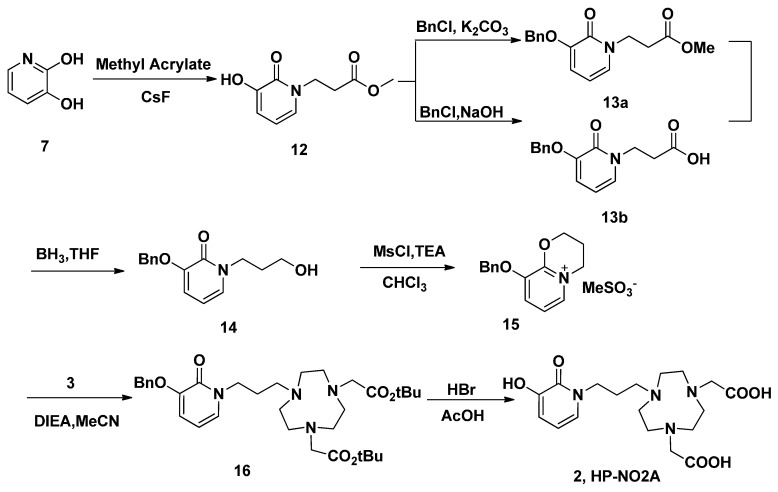
The synthesis of ligand HP-NO2A (**2**).

Similar reaction strategy was applied in the synthesis of HP-NO2A (**2**), since the analogue **2** is just a one carbon extension on the HOPO side arm of **1** (Scheme 4). A Michael addition of methyl acrylate using CsF as a catalyst was employed to introduce a propanoic acid methyl ester side chain on 3,2-HOPO (**7**). The obtained product **12** was reacted with benzyl chloride in a NaOH/MeOH/H_2_O system to form *O*-benzyl protected **13a** in 97% yield after a recrystallization from methanol. The reduction of **13a** using borane-tetrahydrofuran at room temperature obtained **14** in 67% isolated yield. In order to improve the yield, the **14** could also be obtained through the reduction of **13b** using borane-tetrahydrofuran at room temperature in 81% yield, after hydrolysis of **13a**. This reduction method was successful in the preparation of **14** on a larger scale because of the higher yield and easier purification. Following the same conditions used for the synthesis of **4**, alcohol **14** was treated with methanesulfonic chloride in chloroform in the presence of trimethylamine. However, the unusual 3,4-dihydro-2*H*-pyrido[2,1-*b*][1,3]oxazin-5-ium product **15** was isolated. A similar reaction was also reported in another paper [34]. In another report, the electrophilic 3,2-HOPO imidate salt **15**, which can be used for the facile incorporation of the HOPO moiety, reacted with a variety of nucleophiles, including amines [35]. Thus, in the current reaction, alkylation of **3** with **15** in acetonitrile in the presence of DIEA produced **16**. After purification via column chromatography, pure **16** were obtained in 53% yield. The *tert*-butyl and benzyl groups of **16** were then removed with HBr/AcOH to obtain the target compounds HP-NO2A (**2**) in 90% yield.

In conclusion, efficient synthetic routes for two hydroxylpyridinone TACN derivatives have been developed. The new family of monosubstituted macrocyclic ligand HE-NO2A and HP-NO2A with 3,2-HOPO moieties as pendant arms could be used as potential metal chelators in future MRI or PET research. Future study for the ligands in making new Ln(III) complexes (Gd^3+^, Eu^3+^,Tb^3+^,Yb^3+^) and other metal complexes (Cu^2+^, Fe^3+^, Ga^3+^) is in progress.

## 3. Experimental Section

### 3.1. General Information

^1^H- (400 MHz) and ^13^C-NMR (101 MHz) were obtained using a 400 MHz WB Solid-State NMR Spectrometer (Bruker Corporation, Billerica, MA, USA), and chemical shifts are reported in ppm on the δ scale relative to TMS. High-resolution mass spectra (HRMS) were obtained on Bruker ultrafleXtreme MALDI-TOF/TOF matrix:HCCA (Bruker Corporation). 1,4,7-Triazacyclononane was purchased from Synpartner PharmaTech (Quzhou, China). 3,2-Hydroxypridinone was purchased from Aladdin Industrial (Shanghai, China). The other solvents and reagents were obtained from Sinopharm (Shanghai, China). All commercially available reagents were used as received.

### 3.2. 1,4-bis(tert-Butoxycarbonylmethyl)-1,4,7-triazacyclononane *(**3**)*

To a solution of TACN (3.0 g, 23 mmol) in CH_3_CN (50 mL) at 0 °C *t*-butyl bromoacetate (9.0 g, 46 mmol) in CH_3_CN (100 mL) was added dropwise over 4 h. The resulting mixture was allowed to reach room temperature and stirred for 24 h. The reaction mixture was filtered, the filtrate was evaporated, and the residue was treated with DI water (15 mL). The resulting solution was adjusted to pH 3 using 1 M HCl and extracted with ether (3 × 50 mL). The aqueous layer was then adjusted to pH 8 using 1 M NaOH and extracted with CH_2_Cl_2_. The CH_2_Cl_2_ layer was dried, filtered and evaporated to dryness. The residue was treated with ether (50 mL), and the yellowish solid formed and filtered. The solid was treated with hexane (30 mL) and filtered and dried to afford pure **3** (4.0 g, 49%) as a white powder. ^1^H-NMR (CDCl_3_) δ 3.36 (s, 4H), 3.22 (t, *J* = 5.6 Hz, 4H), 3.04 (t, *J* = 5.7 Hz, 4H), 2.77 (s, 4H), 1.46 (s, 18H). ^13^C-NMR (CDCl_3_) δ 170.80, 82.06, 56.63, 51.75, 48.78, 44.66, 28.14.

### 3.3. Ethyl 2-(3-Hydroxy-2-oxopyridin-1(2H)-yl) Acetate *(**8**)*

The mixture of 3,2-hydroxypridinone (5.5 g, 50 mmol) and *tert*-butyl-bromoacetate (25 mL) was heated at 140 °C for 3 days. After cooling to room temperature, the solution was filtered and washed with cold MeOH. Recrystallization the solid from ethanol gave **8** as a white solid (8.4 g, 86%). ^1^H-NMR (DMSO-*d*_6_) δ 9.14 (s, 1H), 7.13 (dd, *J* = 6.9, 1.6 Hz, 1H), 6.72 (dd, *J* = 7.3, 1.6 Hz, 1H), 6.11 (t, *J* = 7.1 Hz, 1H), 4.71 (s, 2H), 4.13 (q, *J* = 7.1 Hz, 2H), 1.30–1.07 (m, 3H). ^13^C-NMR (DMSO-*d*_6_) δ 168.48, 158.35, 147.12, 129.31, 115.81, 105.67, 61.39, 50.76, 14.46.

### 3.4. Ethyl 2-(3-(Benzyloxy)-2-oxopyridin-1(2H)-yl) Acetate *(**9a**)*

To a solution of **8** (1.0 g, 5.1 mmol) in MeCN was added BnCl (1.3 g, 10 mmol) and K_2_CO_3_ (1.4 g, 10 mmol), and the reaction mixture was heated at 50 °C for 4 h. The reaction mixture was filtered and evaporated. The residue was purified by silica column chromatography (ethyl acetate-hexane, 1:3) to obtain **9a** (1.2 g, 84%) as a white solid. ^1^H-NMR (CDCl_3_) δ 7.41 (d, *J* = 7.4 Hz, 2H), 7.34 (t, *J* = 7.3 Hz, 2H), 7.29 (d, *J* = 7.2 Hz, 1H), 6.85 (dd, *J* = 6.9, 1.6 Hz, 1H), 6.66 (dd, *J* = 7.4, 1.5 Hz, 1H), 6.04 (t, *J* = 7.2 Hz, 1H), 5.09 (s, 2H), 4.65 (s, 2H), 4.22 (q, *J* = 7.1 Hz, 2H), 1.37–1.19 (m, 3H). ^13^C-NMR (CDCl_3_) δ 167.69, 158.19, 148.81, 136.19, 129.36, 128.53, 127.97, 127.33, 116.03, 104.92, 70.74, 61.79, 50.48, 14.10.

### 3.5. 2-(3-(Benzyloxy)-2-oxopyridin-1(2H)-yl)acetic Acid *(**9b**)*

Compound **8** (4.6 g, 23 mmol) was dissolved in methanol-water (9:1, 150 mL). To this solution BnCl (11.8 g, 93.2 mmol) and NaOH (2.8 g, 70 mmol) were added, and the mixture was heated to reflux for 6 h. The methanol was removed by evaporation and the aqueous solution was extracted with dichloromethane to remove excess benzyl chloride. The aqueous phase is diluted slightly by adding extra water and then acidified to pH 2 using concentrated hydrochloric acid, which results in the precipitation of a beige solid. The precipitate filtered off and washed with diethyl ether. The crude product was recrystallized from ethanol to give **9b** (5.7 g, 95%) as a white solid. ^1^H-NMR (DMSO-*d*_6_) δ 7.46–7.30 (m, 5H), 7.25 (dd, *J* = 6.9, 1.6 Hz, 1H), 6.92 (dd, *J* = 7.5, 1.5 Hz, 1H), 6.13 (t, *J* = 7.1 Hz, 1H), 5.00 (s, 2H), 4.62 (s, 2H). ^13^C-NMR (DMSO-*d*_6_) δ 169.87, 157.54, 148.36, 136.96, 131.20, 128.86, 128.41, 128.39, 116.19, 104.24, 70.23, 50.66.

### 3.6. 3-(Benzyloxy)-1-(2-hydroxyethyl)pyridin-2(1H)-one *(**10**)*

To a solution of acid **9a** (200 mg, 0.696 mmol) in dry THF (5 mL) under N_2_ was added BH_3_·THF (1.0 M, 1.4 mL, 1.4 mmol), and the reaction mixture was stirred at room temperature for 5 h. The reaction mixture was poured over crushed ice, extracted with dichloromethane, dried (MgSO_4_), and filtered, and the solvents were removed *in* *vacuo* to give **10** (136 mg, 80.0%) as a colorless oil which was used in the next step without purification. ^1^H-NMR (CDCl_3_) δ 7.38 (d, *J* = 7.0 Hz, 2H), 7.34–7.28 (m, 2H), 7.28–7.22 (m, 1H), 6.93 (dd, *J* = 6.9, 1.6 Hz, 1H), 6.64 (dd, *J* = 7.5, 1.5 Hz, 1H), 6.00 (t, *J* = 7.2 Hz, 1H), 5.03 (s, 2H), 4.07 (t, *J* = 4.7 Hz, 2H), 3.88–3.82 (m, 2H), 2.29 (s, 1H). ^13^C-NMR (CDCl_3_) δ 158.99, 148.73, 136.09, 130.05, 128.59, 128.07, 127.38, 116.01, 105.07, 70.77, 61.39, 53.21.

### 3.7. 3-(Benzyloxy)-1-(2-hydroxyethyl)pyridin-2(1H)-one *(**10**)*

To a solution of acid **9b** (5.8 g, 22 mmol) in dry THF (20 mL) under N_2_ was added BH_3_·THF (1.0 M, 44.5 mmol), and the reaction mixture was stirred at room temperature for 5 h. The reaction mixture was poured over crushed ice, extracted with dichloromethane, dried (MgSO_4_), and filtered, and the solvents were removed *in vacuo* to give **10** (2.2 g, 90%) as a colorless oil which was used in the next step without purification.

### 3.8. 3-(Benzyloxy)-1-(2-chloroethyl)pyridin-2(1H)-one *(**4**)*

To a solution of **10** (2.5 g, 10 mmol) and TEA (3.0 g, 30 mmol) in dry CHCl_3_ (30 mL) was added MsCl (1.4 g, 12 mmol) in CHCl_3_ (20 mL) dropwise at 0 °C. The reaction mixture was allowed to warm to room temperature and stirred overnight. The reaction mixture was evaporated and passed a short silica column (ethyl acetate/hexane, 1:5) to give **4** (1.9 g, 72%) as a white solid.^1^H-NMR (CDCl_3_) δ 7.43 (d, *J* = 7.3 Hz, 2H), 7.39–7.33 (m, 2H), 7.33–7.26 (m, 1H), 6.96 (dd, *J* = 6.9, 1.6 Hz, 1H), 6.68 (dd, *J* = 7.5, 1.6 Hz, 1H), 6.04 (t, *J* = 7.2 Hz, 1H), 5.11 (s, 2H), 4.25 (dd, *J* = 7.0, 4.3 Hz, 2H), 3.91 (dd, *J* = 7.0, 4.3 Hz, 2H). ^13^C-NMR (CDCl_3_) δ 158.03, 148.83, 136.13, 130.18, 128.59, 128.04, 127.33, 115.78, 104.37, 70.77, 52.21, 41.91.

### 3.9. tert-Butyl-2,2'-(7-(2-(3-(benzyloxy)-2-oxopyridin-1(2H)-yl)ethyl)-1,4,7-triazonane-1,4-diyl) Diacetate *(**11**)*

To a solution of **3** (357 mg, 1.00 mmol) in acetonitrile (20 mL) was dropwise added DIEA (387 mg, 3.00 mmol) and **4** (316.0 mg, 1.2 mmol) in acetonitrile (10 mL). The reaction mixture was stirred at room temperature (23 °C) for 1 d. After evaporation of the solvent, the residue was purified via silica column chromatography (CH_2_Cl_2_–MeOH, 20:1) to obtain **11** (364 mg, 62.2%) as a yellow oil. ^1^H-NMR (CDCl_3_) δ 7.56–7.47 (m, 1H), 7.44–7.20 (m, 5H), 6.67 (d, *J* = 7.5 Hz, 1H), 6.08 (t, *J* = 7.2 Hz, 1H), 5.02 (s, 2H), 4.51–4.39 (m, 2H), 3.50 (d, *J* = 9.1 Hz, 7H), 3.36 (d, *J* = 5.6 Hz, 2H), 3.22 (d, *J* = 14.3 Hz, 2H), 3.12–2.85 (m, 4H), 2.74 (t, *J* = 20.2 Hz, 2H), 1.40 (s, 18H). ^13^C-NMR (CDCl_3_) δ 169.99, 158.18, 148.49, 135.94, 129.99, 128.58, 128.10, 127.41, 115.94, 105.63, 82.26, 70.74, 56.45, 52.77, 51.60, 50.71, 48.79, 45.46, 28.13. MALDI-HRMS (matrix: HCCA) Calculated for C_32_H_48_N_4_O_6_: [M + H]^+^ *m*/*z* 585.3607, Found 585.3636.

### 3.10. 2,2'-(7-(2-(3-Hydroxy-2-oxopyridin-1(2H)-yl)ethyl)-1,4,7-triazonane-1,4-diyl) Diacetic Acid (HE-NO2A, ***1***)

Compound **11** (100 mg, 0.171 mmol) at 0 °C in 2 mL acetic acid was added dropwise with 37% HBr in acetic acid (5 mL). The resulting mixture was allowed to warm at room temperature and stirred for 2 days. The solvent was evaporated *in vacuo*, the residue was taken up in DI water and passed through a 0.45 μm nylon syringe filter. The aqueous solution was concentrated *in vacuo* to provide HE-NO2A (60 mg, 91%) as a yellow solid. ^1^H-NMR (D_2_O) δ 7.17 (dd, *J* = 6.9, 1.6 Hz, 1H), 7.01 (dd, *J* = 7.6, 1.5 Hz, 1H), 6.47–6.37 (m, 1H), 4.46–4.39 (m, 2H), 3.91 (s, 4H), 3.75–3.69 (m, 2H), 3.61 (s, 4H), 3.45 (t, *J* = 5.6 Hz, 4H), 3.35 (s, 4H). ^13^C-NMR (D_2_O) δ 171.99, 159.43, 145.64, 129.38, 119.55, 109.78, 57.30, 56.77, 51.14, 50.64, 49.52, 47.14. MALDI-HRMS (matrix: HCCA) Calculated for C_17_H_26_N_4_O_6_: [M + H]^+^ *m*/*z* 383.1886. Found: 383.1936.

### 3.11. Methyl 3-(3-Hydroxy-2-oxopyridin-1(2H)-yl) Propanoate *(**12**)*

To a solution of **7** (5.5 g 50 mmol) in methyl acrylate (25 mL) was added CsF (0.5 g), and the reaction mixture was heated at reflux for 2 days. The excess methyl acrylate was removed *in vacuo*. The residue was resolved in hot MeCN, and filtered. The filtratate was evaporated to give the crude, which was recrystallized from ethanol gave **12** as a white solid (8.5 g, 87%). ^1^H-NMR (DMSO-*d*_6_) δ 9.04 (s, 1H), 7.12 (dd, *J* = 6.8, 1.5 Hz, 1H), 6.69 (dd, *J* = 7.2, 1.5 Hz, 1H), 6.08 (t, *J* = 7.0 Hz, 1H), 4.13 (t, *J* = 7.0 Hz, 2H), 3.59 (s, 3H), 2.76 (t, *J* = 6.9 Hz, 2H). ^13^C-NMR (DMSO-*d*_6_) δ 171.64, 158.13, 147.11, 128.85, 115.22, 105.69, 51.97, 45.66, 33.10.

### 3.12. Methyl 3-(3-(Benzyloxy)-2-oxopyridin-1(2H)-yl) Propanoate *(**13a**)*

To a solution of **12** (0.8 g 4 mmol) in MeCN was added BnCl (1.0 g, 7.9 mmol) K_2_CO_3_ (1.1 g, 8.0 mmol), and the reaction mixture was heated at 50 °C for 4 h. The reaction mixture was filtered and evaporated. The residue was purified by silica column chromatography (ethyl acetate–hexane, 1:3) to obtain **13a** (0.9 g, 74%) as a white solid. ^1^H-NMR (CDCl_3_) δ 7.41 (d, *J* = 7.1 Hz, 2H), 7.34 (dd, *J* = 11.4, 4.4 Hz, 2H), 7.31–7.26 (m, 1H), 7.03 (dd, *J* = 6.9, 1.5 Hz, 1H), 6.64 (dd, *J* = 7.4, 1.5 Hz, 1H), 6.00 (t, *J* = 7.2 Hz, 1H), 5.08 (s, 2H), 4.20 (t, *J* = 6.4 Hz, 2H), 3.64 (s, 3H), 2.86 (t, *J* = 6.4 Hz, 2H). ^13^C-NMR (CDCl_3_) δ 171.99, 158.06, 148.76, 136.21, 129.99, 128.54, 127.98, 127.31, 115.61, 104.53, 70.70, 51.85, 46.32, 32.72.

### 3.13. 3-(3-(Benzyloxy)-2-oxopyridin-1(2H)-yl) Propanoic Acid *(**13b**)*

Compound **12** (3.8 g 19 mmol) was dissolved in methanol/water (9:1, 70 mL). To this solution BnCl (9.8 g, 77 mmol) and NaOH (2.4 g, 60 mmol) was added, and the mixture was heated to reflux for 6 h. The methanol was removed by evaporation and the aqueous solution was extracted with dichloromethane to remove excess benzyl chloride. The aqueous phase is diluted slightly by adding extra water and then acidified to pH 2 using concentrated hydrochloric acid, which resulted in the precipitation of a beige solid. The precipitate filtered off and washed with diethyl ether. The crude product was recrystallized from ethanol to give **13b** (5.1 g, 97%) as a white solid. ^1^H-NMR (DMSO-*d*_6_) δ 7.37 (m, 5H), 7.25 (d, *J* = 6.2 Hz, 1H), 6.88 (d, *J* = 7.1 Hz, 1H), 6.10 (t, *J* = 7.1 Hz, 1H), 4.99 (s, 2H), 4.06 (t, *J* = 6.9 Hz, 2H), 2.65 (t, *J* = 6.9 Hz, 2H). ^13^C-NMR (DMSO-*d*_6_) δ 172.76, 157.30, 148.35, 137.00, 130.89, 128.85, 128.41, 128.38, 115.93, 104.29, 70.21, 45.81, 33.29.

### 3.14. 3-(Benzyloxy)-1-(3-hydroxypropyl) Pyridin-2(1H)-one *(**14**)*

To a solution of acid **13a** (2.6 g, 10 mmol) in dry THF (15 mL) under N_2_ was added BH_3_·THF (1 M, 20 mL), and the reaction mixture was stirred at room temperature for 5 h. The reaction mixture was poured over crushed ice, extracted with dichloromethane, dried (MgSO_4_), and filtered, and the solvents were removed *in vacuo* to give **14** (1.5 g, 67%) as a colorless oil which was used in the next step without purification. ^1^H-NMR (CDCl_3_) δ 7.40 (d, *J* = 7.3 Hz, 2H), 7.33 (t, *J* = 7.2 Hz, 2H), 7.30–7.26 (m, 1H), 6.92 (dd, *J* = 6.9, 1.6 Hz, 1H), 6.68 (dd, *J* = 7.5, 1.4 Hz, 1H), 6.10 (t, *J* = 7.1 Hz, 1H), 5.09 (s, 2H), 4.24–4.07 (m, 2H), 3.49 (t, *J* = 5.5 Hz, 2H), 1.95–1.82 (m, 2H). ^13^C-NMR (CDCl_3_) δ 158.94, 148.56, 136.02, 128.83, 128.61, 128.08, 127.37, 115.96, 105.97, 70.80, 57.59, 45.82, 32.33.

### 3.15. 3-(Benzyloxy)-1-(3-hydroxypropyl) Pyridin-2(1H)-one *(**14**)*

To a solution of acid **13b** (2.7 g.10 mmol) in dry THF (20 mL) under N_2_ was added BH_3_·THF (1.0 M, 20 mL, 20 mmol), and the reaction mixture was stirred at room temperature for 5 h. The reaction mixture was poured over crushed ice, extracted with dichloromethane, dried (MgSO_4_), and filtered, and the solvents were removed *in vacuo* to give **14** (2.1 g, 81%) as a colorless oil which was used in the next step without purification.

### 3.16. 9-(Benzyloxy)-3,4-dihydro-2H-pyrido[2,1-b][1,3]oxazin-5-ium Methanesulfonate *(**15**)*

To a solution of **14** (2.6 g, 10 mmol) and TEA (3.0 g, 30 mmol) in dry CHCl_3_ (30 mL) was added dropwise MsCl (1.4 g, 12 mmol) in CHCl_3_ (20 mL) at 0 °C. The reaction mixture was allowed to warm to room temperature and stirred overnight. The reaction mixture was stirred overnight, diluted with CHCl_3_, and washed with 5% NaHCO_3_ (3 × 50 mL) and brine (1 × 50 mL). The organic solution was dried over Na_2_SO_4_ and evaporated *in* *vacuo*. Ethyl acetate (EA) was added to the residue, which resulted in the precipitation of a white solid. The precipitate filtered off and washed with EA to obtain **15** (2.7 g, 80%) as a white solid. ^1^H-NMR (CDCl_3_) δ 8.12 (d, *J* = 6.2 Hz, 1H), 7.69–7.63 (m, 1H), 7.35 (dt, *J* = 8.7, 7.2 Hz, 5H), 7.28–7.21 (m, 1H), 5.18 (s, 2H), 4.85 (dt, *J* = 28.7, 5.5 Hz, 4H), 2.60 (d, *J* = 2.0 Hz, 3H), 2.52–2.37 (m, 2H). ^13^C-NMR (CDCl_3_) δ 161.49, 151.73, 146.13, 134.26, 132.53, 128.87, 127.80, 124.58, 117.84, 72.16, 68.71, 50.59, 39.51, 19.99.

### 3.17. tert-Butyl-2,2'-(7-(3-(3-(benzyloxy)-2-oxopyridin-1(2H)-yl)propyl)-1,4,7-triazonane-1,4-diyl) Diacetate *(**16**)*

To a solution of **3** (357 mg, 1.00 mmol) in acetonitrile was dropwise added DIEA (387 mg, 3.00 mmol) and **15** (400.0 mg, 1.2 mmol) in acetonitrile (10 mL). The reaction mixture was stirred at room temperature for 1 day. After evaporation of the solvent, the residue was purified via silica column chromatography (CH_2_Cl_2_–MeOH, 20:1) to obtain **16** (317.0 mg, 53%) as a yellow oil. ^1^H-NMR (CDCl_3_) δ 7.44–7.20 (m, 6H), 6.67 (d, *J* = 7.4 Hz, 1H), 6.08 (t, *J* = 7.1 Hz, 1H), 5.06 (s, 2H), 4.47 (d, *J* = 6.6 Hz, 2H), 3.54 (s, 3H), 3.47 (s, 4H), 3.38 (d, *J* = 25.6 Hz, 2H), 3.24 (d, *J* = 45.7 Hz, 4H), 2.78 (d, *J* = 24.1 Hz, 4H), 1.43 (s, 18H), 1.26 (dd, *J* = 16.3, 8.6 Hz, 2H). ^13^C-NMR (CDCl_3_) δ 170.44, 158.19, 148.57, 136.00, 129.91, 128.57, 128.07, 127.33, 116.00, 105.53, 81.68, 70.76, 58.46, 54.15, 53.23, 52.57, 51.62, 45.49, 29.64, 28.13. MALDI-HRMS (matrix: HCCA) Calculated for C_33_H_50_N_4_O_6_: [M + H]^+^ *m*/*z* 599.3764, Found 599.3792.

### 3.18. 2,2'-(7-(3-(3-Hydroxy-2-oxopyridin-1(2H)-yl)propyl)-1,4,7-triazonane-1,4-diyl) Diacetic Acid (HP-NO2A, ***2***)

Compound **16** (150 mg, 0.251 mmol) at 0 °C in 2 mL acetic acid was added dropwise with 37% HBr in acetic acid (5 mL). The resulting mixture was allowed to warm to room temperature and stirred for 2 days. The solvent was evaporated *in vacuo*, the residue was taken up in DI water and passed through a 0.45 μm nylon syringe filter. The aqueous solution was concentrated *in vacuo* to provide **2** (89 mg, 90%) as a yellow solid. ^1^H-NMR (D_2_O) δ 7.20 (dd, *J* = 6.8, 1.5 Hz, 1H), 7.02 (dd, *J* = 7.6, 1.6 Hz, 1H), 6.47–6.40 (m, 1H), 4.17–4.07 (m, 2H), 3.95 (s, 3H), 3.99–3.84 (m, 4H), 3.63–3.54 (m, 5H), 3.46 (s, 4H), 3.41–3.30 (m, 6H), 2.40–2.20 (m, 2H). ^13^C-NMR (D_2_O) δ 172.28, 158.73, 145.45, 129.09, 119.66, 109.64, 57.46, 57.07, 54.00, 51.27, 50.17, 49.22, 46.31, 24.09. MALDI-HRMS (matrix: HCCA) Calculated for C_18_H_28_N_4_O_6_: [M + H]^+^ *m*/*z* 397.2042. Found 397.2076.
